# Case report: Discovery of a *de novo FAM111B* pathogenic variant in a patient with an APECED-like clinical phenotype

**DOI:** 10.3389/fimmu.2023.1133387

**Published:** 2023-02-17

**Authors:** Elise M. N. Ferré, Yunting Yu, Vasileios Oikonomou, Anna Hilfanova, Chyi-Chia R. Lee, Lindsey B. Rosen, Peter D. Burbelo, Sara E. Vazquez, Mark S. Anderson, Amisha Barocha, Theo Heller, Ariane Soldatos, Steven M. Holland, Magdalena A. Walkiewicz, Michail S. Lionakis

**Affiliations:** ^1^ Laboratory of Clinical Immunology and Microbiology (LCIM), National Institute of Allergy and Infectious Diseases (NIAID), National Institutes of Health (NIH), Bethesda, MD, United States; ^2^ Division of Intramural Research, National Institute of Allergy and Infectious Diseases (NIAID), National Institutes of Health (NIH), Bethesda, MD, United States; ^3^ Department of Pediatrics, Immunology, Infectious and Rare Diseases, Medical School of the International European University, Kyiv, Ukraine; ^4^ Laboratory of Pathology, Clinical Center for Cancer Research, National Cancer Institute, National Institutes of Health (NIH), Bethesda, MD, United States; ^5^ National Institute of Dental and Craniofacial Research, National Institutes of Health (NIH), Bethesda, MD, United States; ^6^ Diabetes Center, University of California, San Francisco, San Francisco, CA, United States; ^7^ Laboratory of Asthma and Lung Inflammation, National Heart Lung and Blood Institute (NHLBI), National Institutes of Health (NIH), Bethesda, MD, United States; ^8^ Liver Diseases Branch, National Institute of Diabetes and Digestive and Kidney Disease, National Institutes of Health, Bethesda, MD, United States; ^9^ National Institute of Neurological Disorders and Stroke (NINDS), National Institutes of Health (NIH), Bethesda, MD, United States

**Keywords:** Primary immunodeficiency disorders, Autoimmunity, POIKTMP, FAM111B, Chronic mucocutaneous candidiasis, immunosuppression

## Abstract

**Introduction:**

Autoimmune polyendocrinopathy-candidiasis-ectodermal dystrophy (APECED) and poikiloderma in association with tendon contractures, myopathy, and pulmonary fibrosis (POIKTMP) are rare inherited syndromes resulting from biallelic pathogenic variants in *AIRE* and heterozygous pathogenic variants in *FAM111B*, respectively. The clinical diagnosis of APECED and POIKTMP rely on the development of two or more characteristic disease manifestations that define the corresponding syndromes. We discuss the shared and distinct clinical, radiographic, and histological features between APECED and POIKTMP presented in our patient case and describe his treatment response to azathioprine for POIKTMP-associated hepatitis, myositis, and pneumonitis.

**Methods:**

Through informed consent and enrollment onto IRB-approved protocols (NCT01386437, NCT03206099) the patient underwent a comprehensive clinical evaluation at the NIH Clinical Center alongside exome sequencing, copy number variation analysis, autoantibody surveys, peripheral blood immunophenotyping, and salivary cytokine analyses.

**Results:**

We report the presentation and evaluation of a 9-year-old boy who was referred to the NIH Clinical Center with an APECED-like clinical phenotype that included the classic APECED dyad of CMC and hypoparathyroidism. He was found to meet clinical diagnostic criteria for POIKTMP featuring poikiloderma, tendon contractures, myopathy, and pneumonitis, and exome sequencing revealed a *de novo* c.1292T>C heterozygous pathogenic variant in *FAM111B* but no deleterious single nucleotide variants or copy number variants in *AIRE*.

**Discussion:**

This report expands upon the available genetic, clinical, autoantibody, immunological, and treatment response information on POIKTMP.

## Introduction

Early-onset poikiloderma in association with tendon contractures, myopathy, and pulmonary fibrosis (POIKTMP) is a rare, autosomal-dominant syndrome caused by *FAM111B* variants (1–3). Autoimmune polyendocrinopathy-candidiasis-ectodermal dystrophy (APECED) or autoimmune polyglandular syndrome type-1 (APS-1) features AIRE deficiency that causes impaired thymic negative selection of T-lymphocytes and multiorgan autoimmunity (4, 5). The clinical diagnosis of APECED relies on developing any two manifestations among the classic triad of chronic mucocutaneous candidiasis (CMC), hypoparathyroidism, and adrenal insufficiency (6, 7). Although POIKTMP and APECED are distinct entities, we describe the clinical, radiographic, immunological, autoantibody, and genetic evaluation of a patient referred to the NIH with an APECED-like clinical disease, in whom we identified a novel, *de novo* heterozygous missense variant in *FAM111B* (c.1292T>C, p.Phe431Ser) and discuss the phenotypic overlap and differences between these two monogenic syndromes.

## Case report

**Figure 1:**
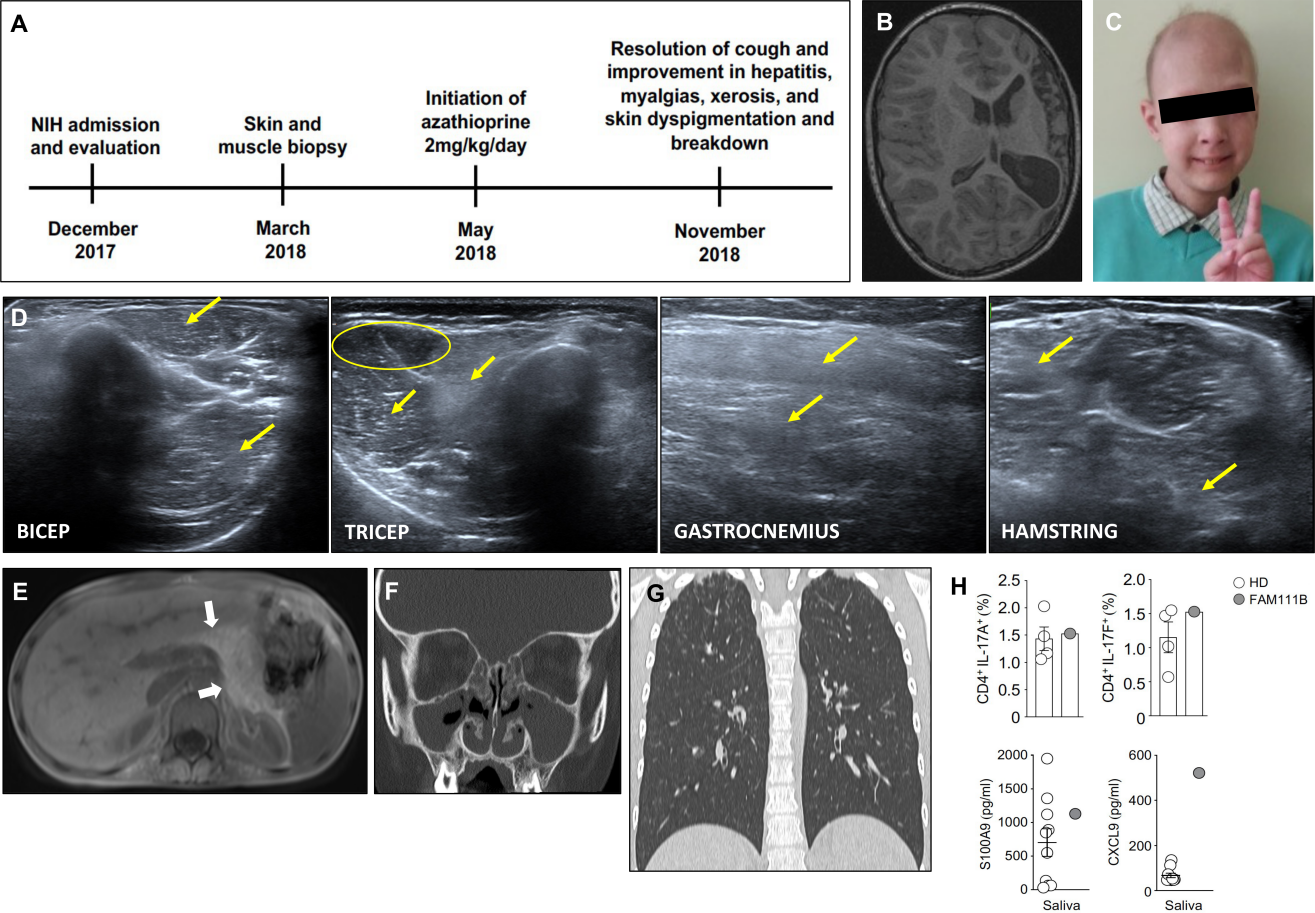
Clinical and radiographic features observed in our patient. **(A)** Timeline of the patient’s evaluation and treatment course. **(B)** Axial cut from T1-weighted MRI brain showing striking hemiatrophy of the entire left hemisphere, presumably due to the hemorrhagic stroke in the neonatal period. **(C)** Photograph of our patient at the time of evaluation at the NIH demonstrating patchy alopecia with sparse brittle hair as well as poikiloderma and conical fingers, which are characteristic of POIKTMP. **(D)** Muscle ultrasound revealing myositis evidenced by diffusely hyperechoic muscles in the upper and lower extremities. Yellow arrows point to increased echogenicity of muscle compared to a less echogenic area (yellow circle). **(E)** Abdominal MRI revealing complete fatty replacement of the pancreas (white arrows) consistent with the clinical history of exocrine pancreatic insufficiency. **(F)** CT of sinuses showing complete opacification of bilateral maxillary and ethmoid sinuses. **(G)** Coronal (right panel) view of non-contrast chest CT revealing bilateral ground glass opacities in the apices and scattered tree-in-bud nodular opacities throughout bilateral upper and lower lobes. **(H)** Upper panels show the frequencies of IL-17A^+^ (left) and IL-17F^+^ (right) producing cells within CD4^+^ T cells in the peripheral blood from our patient and four healthy donors. The lower panels display levels of salivary S100A9 (left) and CXCL9 (right) from our patient and 10 healthy donors.

A 9-year-old Ukrainian male presented at the NIH for clinical and genetic evaluation of potential APECED (**Figure 1A**). He was born full-term to nonconsanguineous parents in Ukraine. His father has type-2 diabetes, his younger brother is healthy, and his mother died from pancreatic cancer. (**Supplemental Figure 1**). At birth he had microcephaly and jaundice followed by epistaxis and gastric bleeding in the early neonatal period. At 1.5-months-old, he developed cerebral hemorrhage leading to transient left hemiparesis and encephalomalacia (**Figure 1B**); hypoplasia of the right vertebral artery and the left middle cerebral artery (MCA) was noted with compensatory doubling of the right MCA. Alopecia and poikiloderma began at 6-weeks of age progressing within 12-months to loss of scalp hair, eyebrows, and eyelashes and facial skin changes. At 2-months-old, transaminitis with a negative infectious evaluation raised suspicion for autoimmune hepatitis. Recurrent ear and sinopulmonary infections began at 3-months of age requiring antibiotics. At 2-years-old, he developed non-bloody diarrhea with bloating and foul-smelling flatulence that resolved with pancreatic enzyme replacement at 4-years-old. At 6-years-old, he developed hypothyroidism and hypoparathyroidism. Review of outside medical records revealed a constellation of ophthalmologic, oral, and dermatologic manifestations occurring throughout childhood including recurrent blepharoconjunctivitis, chronic erythema and cracking of perioral skin consistent with *Candida* cheilitis that responded to antifungal therapy, dry mouth and eyes, dysphagia, recurrent aphthous stomatitis, gum abscesses, decalcified enamel with several dental caries, hyperkeratosis and xerosis of bilateral palms and soles, anhidrotic ectodermal dysplasia with impaired thermoregulation, kyphosis, arthritis of the hips and ankles, bipedal edema, iron deficiency anemia, and vitamin K-dependent coagulopathy. He also displayed cognitive and speech developmental delays in early childhood.

The combination of hypoparathyroidism and chronic *Candida* cheilitis met the classic diagnostic APECED criteria, which prompted AIRE sequencing that identified a benign, common homozygous variant (c.834C>G, p.Ser278Arg), which was recently found to be enriched in patients with an APECED-like phenotype suffering from autoimmunity (8). He was referred to our APECED natural history study for further evaluation (**Figure 1A**). At NIH admission, he had persistent cough and chronic myalgias of calves and thighs exacerbated by ambulation. He appeared thin with alopecia totalis, erythematous, dry, and hyper/hypopigmented skin with conical-shaped fingers (**Figure 1C**), and non-pitting edema in bilateral feet and ankles. He met Sjogren’s syndrome diagnostic criteria with markedly decreased salivary flow rate (0.06534 ml/15 minutes) (9). He had enamel hypoplasia, congenital absence of several teeth with short, tapered roots on all permanent teeth, a high-arched palate and cheilitis with *Candida albicans*, which resolved with a 4-week course of fluconazole. 

Laboratory evaluations confirmed hypoparathyroidism (calcium, 1.81mmol/dl; intact parathyroid hormone, 16.5 pg/ml). He was euthyroid (thyroid stimulating hormone, 2.5 mcIU/mL; free thyroxine, 1.6 ng/dL) while receiving levothyroxine. An ACTH stimulation test revealed intact adrenal function and 21-hydroxylase autoantibodies were negative. Aspartate aminotransferase (AST; 73 U/L) and alanine aminotransferase (ALT; 146 U/L) were elevated while classical autoimmune hepatitis autoantibodies (i.e., anti-mitochondrial, anti-smooth muscle, anti-liver-kidney microsomal, anti-nuclear) were negative, a pattern that can be observed in APECED-associated hepatitis (10). Given his age and safety concerns due to history of bleeding, a liver biopsy was deferred. Needle electromyogram (EMG) and nerve conduction studies identified small polyphasic units, mild muscle irritability, and sensory neuropathy, whereas muscle ultrasound showed diffusely hyperechoic muscles (**Figure 1D**). These findings together with decreased muscle mass and elevated erythrocyte sedimentation rate (38 mm/hr), creatine kinase (310 U/L), and aldolase (9.5 U/L) were consistent with autoimmune myopathy and sensory neuropathy.

Abdominal MRI demonstrated complete fatty replacement of the pancreas (**Figure 1E**) which combined with undetectable fecal pancreatic elastase-1 confirmed exocrine pancreatic insufficiency. Computed tomography (CT) showed near complete opacification of bilateral maxillary and ethmoid sinuses (**Figure 1F**) and cultures grew *Staphylococcus aureus* and *Streptococcus pyogenes* for which he received amoxicillin/clavulanate. CT chest revealed bilateral pulmonary infiltrates in the apices and right lower lobe with scattered small nodules (**Figure 1G**; **Supplemental Figure 2**). He walked 459 meters and did not desaturate on a 6-minute walk test. Pulmonary function testing (PFT) was limited due to patient’s inability to maintain good quality breath hold thus lung volumes and diffusion capacity could not be measured, but spirometry values were within normal limits without evidence of obstruction or restriction. BPIFB1 and KCNRG autoantibodies, seen in APECED pneumonitis (11) were not detected. Bronchoscopy showed thick, white secretions throughout the bronchial tree with airway neutrophil expansion (73% of nucleated cells). Endobronchial biopsies were not performed due to safety concerns. His symptoms, radiographic abnormalities, and airway neutrophilia were reminiscent of APECED-associated pneumonitis (11).

We performed blood lymphocyte immunophenotyping and additional autoantibody surveys. Lymphocyte subsets were within normal range except for increased CD21^lo^CD38^lo^ B-lymphocytes (**Supplemental Table 1**). He did not carry autoantibodies against Th17 cytokines or type-1 interferons (IFN), including IFN-ω, that are common in APECED (12, 13) (**Supplemental Table 2**) nor did a proteome-wide search for tissue-specific autoantibodies by Phip-Seq identify targets shared with APECED patients (14). Among the identified Phip-Seq autoantibody targets with the highest z-scores relative to healthy controls, six (i.e., CRTAC1, HTRA1, SHANK2, TSHZ3, IVL, TAGAP) are expressed in pancreas, lung, liver, skin, and oral epithelium correlating with the patient’s organ-specific manifestations (**Supplemental Table 3**). Given his history of CMC and the importance of IL-17 responses in mucosal anti-*Candida* defense, we assessed his proportion of circulating Th17 cells and found them normal (**Figure 1G**; **Supplemental Figure 3**). Moreover, the IL-17-dependent antimicrobial peptide, S100A9, was within the normal range in his saliva (**Figure 1H**). We recently reported that enhanced mucosal type-1 responses can promote mucosal fungal susceptibility in the setting of autoimmunity *via* promoting epithelial barrier disruption (13). Of note, although his circulating T-lymphocytes did not produce more IFN-γ upon PMA/ionomycin stimulation (**Supplemental Figure 3B**), the IFN-γ-inducible chemokine, CXCL9, was increased in the saliva (**Figure 1H**).

**Figure 2:**
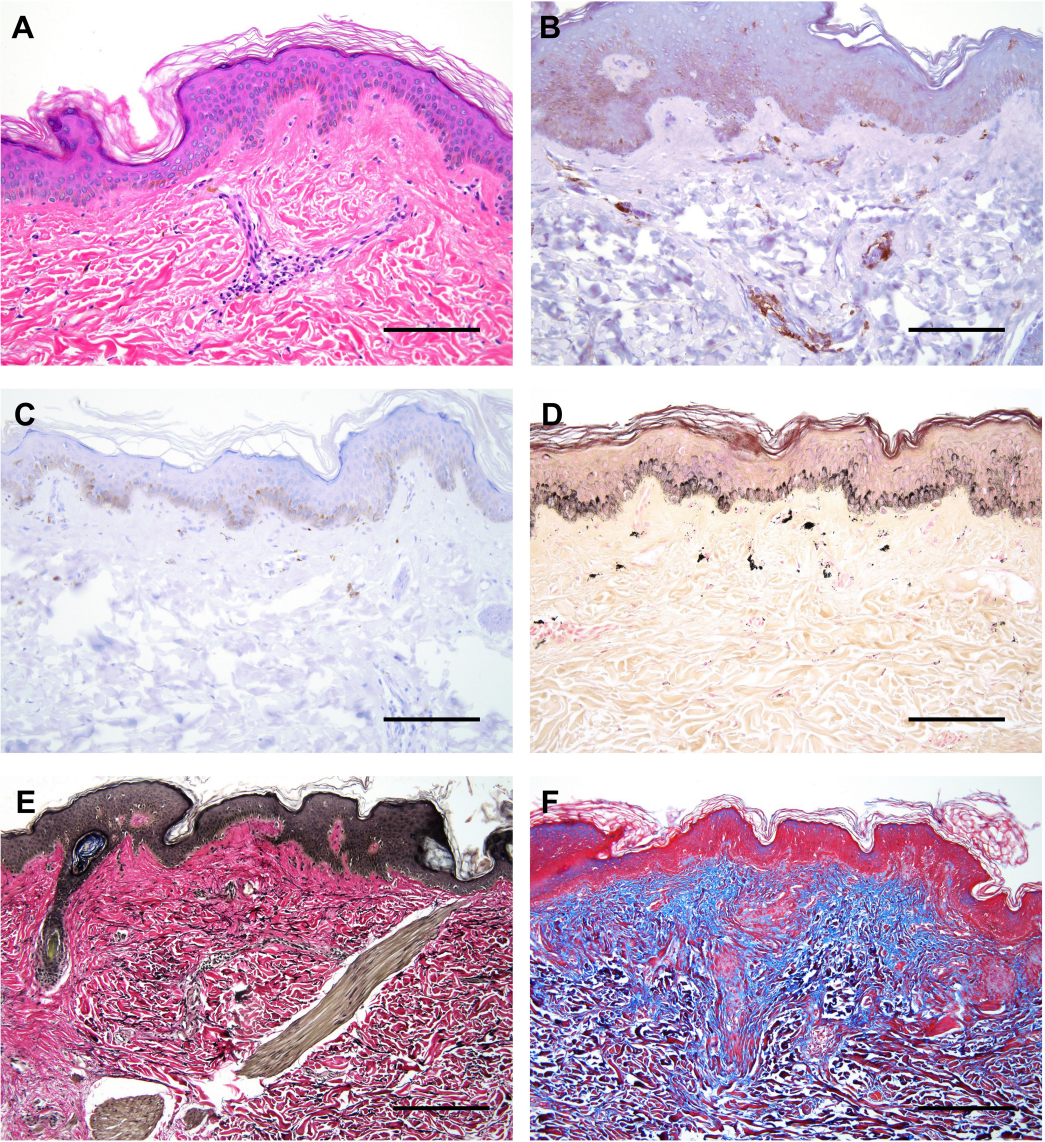
Histological features of affected skin in our patient. **(A)** H&E staining showing mild acanthosis, superficial perivascular lymphocytic infiltration, and pigmentary incontinence. **(B)** Double immunostaining with CD4 (brown) and CD8 (red) revealing focal presence of CD4^+^ T lymphocytes surrounding superficial dermal vasculature. **(C)** Immunostaining with CD20 depicting very few to rare B lymphocytes. **(D)** Fontana-Masson stain highlighting melanin pigments and melanophages in the superficial dermis, termed ‘pigmentary incontinence’. **(E)** EVG (Verhoff Van Gieson) stain highlighting focally increased density of elastic fibers in the reticular dermis, corresponding to the region of solar elastosis. **(F)** Masson’s Trichrome stain highlighting mild expansion of the papillary dermal collagen zone (blue areas). All scale bars correspond to 600 μm.

**Figure 3:**
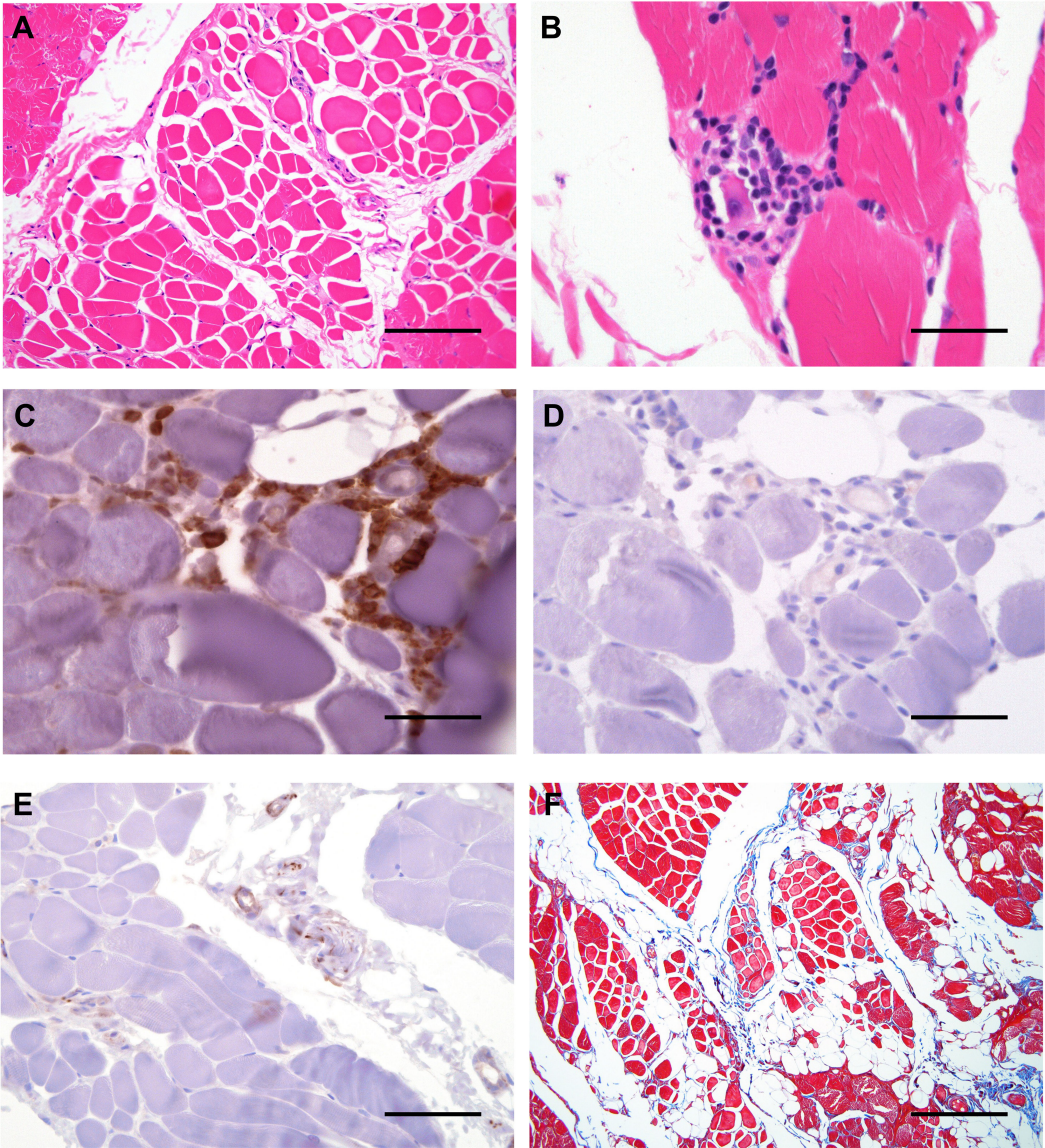
Histological features of affected muscle in our patient. **(A)** Cross sections of H&E staining of skeletal muscle revealing variation in the diameter/caliber of the skeletal muscle fibers with focal infiltrate of the mature adipose tissue. **(B)** High magnification view of H&E staining of skeletal muscle revealing focal lymphocytic infiltrate surrounding a degenerate muscle fiber, consistent with myositis. **(C)** Double immunostaining with CD4 (brown) and CD8 (red) depicting lymphocytic infiltration by CD4^+^ T lymphocytes. **(D)** Immunostaining with CD20 showing no B lymphocyte infiltration. **(E)** Immunostaining with CD56 staining depicting CD56^+^ N-CAM fibers. **(F)** Masson-Trichrome stain highlighting focally increased collagen between the smooth muscle bundles. Scale bars, 2 μm **(A, E)**, 6 μm **(F)** or 200 μm **(B–D)**.

Skin punch and muscle biopsies were performed on the left lower extremity. Skin histological examination revealed mild superficial perivascular chronic inflammation in the dermis (**Figure 2A**) primarily composed of CD4^+^ T-lymphocytes (**Figures 2B, C**). Scattered pigmented melanin-containing macrophages were seen in the superficial dermis (**Figure 2D**) consistent with pigmentary incontinence, which can occur in post-inflammatory hyper- or hypopigmentation. There was focally increased density of elastic fibers in the reticular dermis (**Figure 2E**) and expansion of papillary dermal collagen (**Figure 2F**), consistent with histologic features of solar elastosis. Muscle histological examination revealed focal areas of skeletal muscle fibers arranged in a predominately myopathic pattern associated with focal fatty infiltration (**Figure 3A**). A focal lymphocytic infiltrate surrounding degenerate muscle fibers was noted, consistent with myositis (**Figure 3B**). The inflammatory infiltrate consisted of CD4^+^ T-lymphocytes without CD8^+^ T- or B-lymphocytes (**Figures 3C, D**). Focally increased CD56^+^ N-CAM fibers were noted (**Figure 3E**) as well as slightly increased collagen between the smooth muscle bundles (**Figure 3F**).

**Figure 4:**
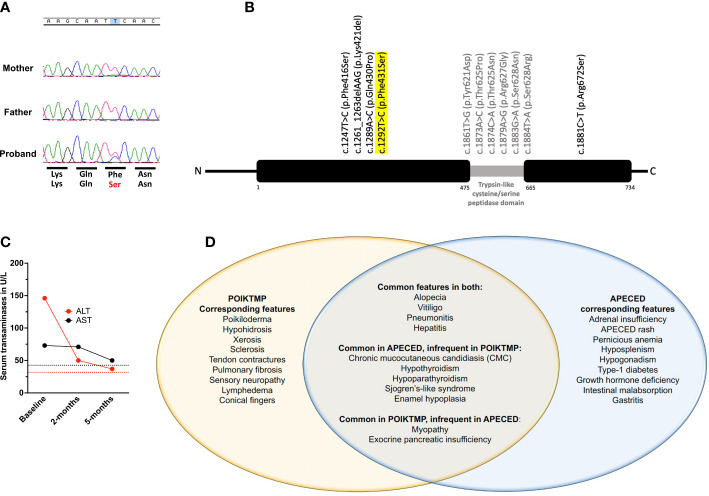
Genetic, clinical, and treatment response characteristics of our patient. **(A)** Chromatograms of *FAM111B* sequencing on our patient and his mother and father shown over corresponding normal amino acid sequence (top row) with patient’s missense change displayed in red (bottom row). **(B)** Schematic of the *FAM111B* gene demonstrating all previously reported variants and their location along with our patient’s variant that is highlighted in yellow. **(C)** Temporal evolution of serum transaminase levels. ALT (red) and AST (black) are increased at baseline and decline nearly to normal levels by 5-months following initiation of azathioprine treatment. Red and black dotted lines represent the upper limit of normal range for ALT and AST, respectively. **(D)** Venn diagram depicting our patient’s disease manifestations, thereby demonstrating the shared and distinct clinical features between POIKTMP and APECED syndromes.

Exome sequencing (ES) identified a novel, heterozygous missense variant (c.1292T>C, p.Phe431Ser) in *FAM111B* [Chr11:58892862 (GRCh37) NM_198947], which was confirmed by Sanger sequencing (**Figures 4A, B**). No copy number variants (CNVs) were identified at the *FAM111B* locus, but a copy number loss was noted within chromosome band 6p25.2 spanning approximately 0.006 Mb in a non-disease associated region involving the *SERPINB9* gene. His parents underwent targeted Sanger sequencing. Neither parent carried the *FAM111B* variant, thereby this was a *de novo* change in the patient (**Figure 4A**). ES and CGH did not identify rare deleterious *AIRE* single nucleotide variants nor CNVs. Moreover, ES did not identify pathogenic or likely pathogenic variants in genes associated with autoimmunity.

APECED-associated hepatitis and pneumonitis respond to azathioprine (AZA)-based treatment (10, 11). He had normal thiopurine methyltransferase activity and was started on AZA at 2mg/kg/day. Within 6 months, cough resolved, transaminases decreased (**Figure 4C**), and skin dyspigmentation, xerosis, skin breakdown, and myalgias improved with an associated decrease in creatine kinase (270 U/L). Eighteen months following AZA initiation, transaminases were normal, but he developed leukopenia (3.4 k/μL) and normocytic anemia (Hgb, 10.7 g/dL), prompting AZA discontinuation with resolution of hematological abnormalities.

## Discussion

We present a 9-year-old boy who met APECED diagnostic criteria in whom a detailed clinical evaluation and ES helped establish a POIKTMP diagnosis. Pathogenic heterozygous missense FAM111B variants resulting in POIKTMP have been reported in 36 patients, with about half being *de novo* as in our patient (15–25). *FAM111B* encodes a homologous serine protease whose function is poorly-understood. Recent studies suggest that *FAM111B* mutations may cause a gain-of-function in the protease’s proteolytic activity that affects cellular fitness by impairing DNA and RNA synthesis, disrupting microtubule network integrity, and triggering caspase-dependent apoptosis (16). The disruption in these cellular processes is thought to promote the fibrotic and chronic inflammatory sequalae of POIKTMP and contribute to the development of certain malignancies, including pancreatic, prostate, and lung adenocarcinomas (3, 26). Although we did not directly demonstrate experimentally that the c.1292T>C, p.Phe431Ser FAM111B variant is dysfunctional, this is supported by strong genetic evidence as outlined in the ACMG guidelines (18) thereby meeting the following criteria: 1) it is *de novo*, as it was confirmed to be absent in both parents, 2) the variant is absent in healthy controls at gnomAD (https://gnomad.broadinstitute.org), and 3) the variant is within one codon of another pathogenic FAM111B variant, p.Gln430Pro, which was previously reported in a patient suffering from POIKTMP (19).

POIKTMP patients exhibit distinctive early-onset dermatologic abnormalities (poikiloderma, alopecia) in association with tendon contractures, progressive muscle weakness, and pulmonary fibrosis. However, 26 other clinical features have been described among the reported 36 patients (19) indicating that the phenotype of POIKTMP is broader than the classic clinical features alone (18). Our patient had both classic features of POIKTMP and non-classic disease manifestations including CMC, hypoparathyroidism, enamel hypoplasia, and Sjogren’s-like syndrome which had not previously been reported in POIKTMP (**Figure 4D**) as well as hypothyroidism, which was reported in a single patient carrying the c.1289A>C *FAM111B* variant.

The classic diagnostic dyad of CMC and hypoparathyroidism had initially raised suspicion for APECED (12). His other clinical manifestations of hypothyroidism, Sjogren’s-like syndrome, pneumonitis, hepatitis, enamel hypoplasia, exocrine pancreatic insufficiency, and myopathy can also occur in APECED with varying frequencies (10, 11, 20). APECED also affects the skin causing alopecia and vitiligo, both observed in our patient, as well as nail dystrophy and early-onset urticarial eruption, termed APECED rash, which we have proposed to include alongside enamel hypoplasia and intestinal malabsorption in expanded diagnostic criteria that may accelerate APECED diagnosis (7, 12).

Although APECED and POIKTMP share some clinical features (**Figure 4D**), distinct clinical and histological characteristics clearly differentiate them. APECED-associated hepatitis features lymphoplasmacytic inflammation with or without peri-portal fibrotic expansion whereas POIKTMP-associated liver disease features macro- and/or micro-vesicular steatosis with or without lymphocytic peri-portal inflammation and fibrosis (19, 21). APECED-associated pneumonitis features decreased diffusion lung capacity, an obstructive, restrictive, or mixed pattern on spirometry, and compartmentalized immunopathology with airway neutrophil expansion and lymphocytic inflammation within epithelial, submucosal, interstitial, and bronchiolar areas (11, 22). POIKTMP-associated pneumonitis features decreased diffusion lung capacity, a restrictive spirometry defect, and histological evidence of interstitial pneumonitis and bronchiolitis with pronounced fibrosis which is not prominent in APECED pneumonitis (23, 24).

Myopathy and poikiloderma are well-described in POIKTMP. Fatty infiltration of the muscle is apparent on MRI with a myogenic pattern on EMG and elevated creatine kinase (18, 25). Histologically, fatty infiltration, dystrophic myocytes with focal lymphocytic infiltration, necrosis, and degeneration are seen. N-CAM (CD56)-positive fibers are typically enriched within dystrophic areas (18, 25). A single patient with APECED-associated myopathy had progressive weakness and muscle wasting with normal muscle MRI and a myogenic pattern on EMG (20). Muscle biopsy in our patient showed marked variation in fiber size with necrosis and degenerative features and focal lymphocytic infiltration. Skin biopsy can also help distinguish POIKTMP from APECED. Our patient’s skin displayed classical features of POIKTMP including hyperkeratosis and melanin deposition in the papillary dermis with lymphocytic infiltration and thickened elastic fibers (25, 27). By contrast, a mixed inflammatory infiltrate comprising both lymphoid and myeloid cells characterizes the APECED rash (12).

There is scarce published information on the management of POIKTMP. Among the reported 36 patients, one had resolution of poikiloderma and hepatitis with corticosteroids and AZA. Another patient with lymphocytic pneumonitis was treated with corticosteroids with little effect and succumbed to his disease (24). In our patient, AZA provided sustained improvement of skin abnormalities, myopathy, transaminitis, and chronic cough before he developed AZA-associated hematological toxicity.

Our patient expands the clinical spectrum of POIKTMP to include CMC, enamel hypoplasia, hypoparathyroidism, and Sjogren’s-like syndrome and highlights the overlap of several disease manifestations with APECED, including the aforementioned diseases, and hypothyroidism, exocrine pancreatic insufficiency, hepatitis, and pneumonitis, which may be ameliorated by lymphocyte-directed immunomodulation. This case additionally highlights the clinical and diagnostic challenges in patients presenting with APECED-like clinical features who do not carry pathogenic AIRE mutations, many of whom remain undiagnosed. Here, ES was critical in establishing a genetic diagnosis *via* identifying a *de novo* FAM111B variant. Phip-Seq-based autoantibody profiling of additional POIKTMP patients is needed to discern whether the identified autoantigen targets in our patient are private or broadly shared, and further immunophenotyping of T lymphocytes, including of regulatory T cells, will be needed to better characterize the immunopathogenesis of POIKTMP. The absence of Th17 cytokine-targeted autoantibodies and the normal circulating Th17 cells and salivary S100A9 in our patient indicate that CMC in POIKTMP may occur in the setting of intact type-17 responses. The increased salivary CXCL9 in our patient suggests that enhanced mucosal type-1 responses may contribute to CMC in POIKTMP, which will require investigation in mice and additional patients with POIKTMP. Moving forward, systematic genetic and clinical evaluation and longitudinal follow-up of several POIKTMP patients will be needed to define the full spectrum of the disease phenotype and to further characterize the safety and efficacy of AZA-based treatment.

## Data availability statement

The original contributions presented in the study are included in the article/**Supplementary Materials**. Further inquiries can be directed to the corresponding authors. 

## Ethics statement

The studies involving human participants were reviewed and approved by National Institute of Allergy and Infectious Diseases IRB board. Written informed consent to participate in this study was provided by the participants’ legal guardian/next of kin. Written informed consent was obtained from the minor(s)’ legal guardian/next of kin for the publication of any potentially identifiable images or data included in this article.

## Author contributions

EF, YY, MW, and ML contributed to the conception and design of the study. EF, ML AH, MA, TH, and AS took care of the patient during the course of the study. C-CL performed immunohistochemical analysis of patient tissues. VO, LR, PB, SV and MA performed experiments with patient’s sera and PBMCs. EF wrote the first draft of the manuscript. SH and ML provided supervision over the project. All authors contributed to the article and approved the submitted version. 
