# Supplementary material for: Case report: Discovery of a de novo FAM111B pathogenic variant in a patient with an APECED-like clinical phenotype
Source: Front Immunol. 2023 Feb 17;14:1133387. doi: 10.3389/fimmu.2023.1133387 (PMC9981804; doi:10.3389/fimmu.2023.1133387)
Supplement: Supplementary file 1 [file DataSheet_1.docx]

Supplementary Material and Methods

A case of mistaken identity: Discovery of a *de novo FAM111B* pathogenic variant in a patient with an APECED-like clinical phenotype

Elise M. N. Ferré^1†^, Yunting Yu^2†^, Vasileios Oikonomou^1^, Anna Hilfanova^3^, Chyi-Chia R. Lee^4^, Lindsey B. Rosen^1^, Peter D. Burbelo^5^, Sara E. Vazquez^6^, Mark S. Anderson^6^, Amisha Barocha^7^, Theo Heller^8^, Ariane Soldatos^9^, Steven M. Holland^1^, Magdalena A. Walkiewicz^2¶^, Michail S. Lionakis^1¶*^

*** Correspondence:** Michail S. Lionakis, MD, ScD: [lionakism@mail.nih.gov](mailto:lionakism@mail.nih.gov)

# Supplementary Methods

The patient was enrolled onto IRB-approved protocols (NCT01386437, NCT03206099) after obtaining informed consent, and underwent a comprehensive APECED evaluation at the NIH Clinical Center.(1, 2) Exome sequencing (ES) was performed on an Illumina sequencing system (minimum coverage, >95% at 20X). Variant interpretation was performed according to ACMG guidelines,(3).

A custom comparative genome hybridization (CGH) array included 2408 genes involved in immunity (Gene Ontology terms containing immun*) and noncoding exons from 15 RNA genes or pseudogenes. Overall, >99% of the targeted exons is covered by at least 3-4 probes and each of the 2408 genes had one probe approximately every 10 kb, while the entire genome backbone had one probe approximately every 55 kb, thus enabling copy number variation analysis at the individual exon or gene level and genome-wide.

A particle-based approach was used to detect immunoreactivity against cytokines and interferons.(1) Luciferase immunoprecipitation systems measured autoantibodies against the lung antigens bacterial/permeability-increasing fold-containing B1 (BPIFB1) and potassium channel regulatory (KCNRG).^(4)^ Proteome-wide autoantibody detection was performed via phage immunoprecipitation sequencing (PhIP-Seq).^(5)^ Lymphocyte immunophenotyping was performed in frozen peripheral blood mononuclear cells (PBMC).(1, 4) PBMC were stimulated with PMA/ionomycin and IL-17A, IL-17F, IL-22, and IFN-γ production by CD4^+^ and CD8^+^ T-lymphocytes was assessed via intracellular staining with flow cytometry as previously described(6). The flow cytometric gating strategy is shown in Supplemental Figure 4. Salivary levels of S100A9 and CXCL9 were measured using ELISA as previously described(6).

1. Ferre EM, Rose SR, Rosenzweig SD, Burbelo PD, Romito KR, Niemela JE, et al. Redefined clinical features and diagnostic criteria in autoimmune polyendocrinopathy-candidiasis-ectodermal dystrophy. JCI Insight. 2016;1(13).

2. Similuk MN, Yan J, Ghosh R, Oler AJ, Franco LM, Setzer M, et al. Clinical Exome Sequencing of 1000 Families with Complex Immune Phenotypes: Towards comprehensive genomic evaluations. J Allergy Clin Immunol. 2022.

3. Richards S, Aziz N, Bale S, Bick D, Das S, Gastier-Foster J, et al. Standards and guidelines for the interpretation of sequence variants: a joint consensus recommendation of the American College of Medical Genetics and Genomics and the Association for Molecular Pathology. Genet Med. 2015;17(5):405-24.

4. Ferre EMN, Break TJ, Burbelo PD, Allgauer M, Kleiner DE, Jin D, et al. Lymphocyte-driven regional immunopathology in pneumonitis caused by impaired central immune tolerance. Sci Transl Med. 2019;11(495).

5. Vazquez SE, Ferre EM, Scheel DW, Sunshine S, Miao B, Mandel-Brehm C, et al. Identification of novel, clinically correlated autoantigens in the monogenic autoimmune syndrome APS1 by proteome-wide PhIP-Seq. Elife. 2020;9.

6. Break TJ, Oikonomou V, Dutzan N, Desai JV, Swidergall M, Freiwald T, et al. Aberrant type 1 immunity drives susceptibility to mucosal fungal infections. Science. 2021;371(6526).

# Supplementary Data

**Supplemental Table 1: Peripheral blood lymphocyte subsets in our patient with POIKTMP.**

|  | | **OUR PATIENT** | | **ADULT HEALTHY CONTROLS (95% CI)** | |
| --- | --- | --- | --- | --- | --- |
|  |  | **%within lymphocytes** | **Absolute number** | **% within lymphocytes** | **Absolute number** |
| **T cells** | CD3 | 73.8% | 1225 | **60.0-83.7%** | **714-2266** |
|  | CD3/alpha beta | 69.5% | 1154 | **55.8-80.1%** | **675-2235** |
|  | CD3/gamma delta | 4.4% | 73 | **0.5-10.4%** | **8-261** |
|  |  |  |  |  |  |
| **T cell subsets** | CD4/CD3 | 45.2% | 750 | **31.9-62.2%** | **359-1565** |
|  | CD8/CD3 | 22.9% | 380 | **11.2-34.8%** | **178-853** |
|  | T4/T8 ratio | 1.97 |  | **1.11-5.17** |  |
|  | CD3+/CD4-/CD8- | 5.4% | 90 | **1.3-9.2%** | **18-185** |
|  | CD4/CD3/CD62L+/CD45+ | 26.3% | 437 | **7.6-37.7%** | **102-1041** |
|  | CD4/CD3CD62L+/CD45- | 14.0% | 232 | **10.4-30.7%** | **162-614** |
|  | CD4/CD3/CD62L-/CD45RA- | 4.6% | 76 | **2.3-15.6%** | **42-225** |
|  | CD4/CD3/CD62L-/CD45RA+ | 0.3% | 5 | **0-1.5%** | **0-29** |
|  | CD8/CD3/CD62L+/CD45RA+ | 11.8% | 196 | **5.7-19.7%** | **85-568** |
|  | CD8/CD3/CD62L+/CD45RA- | 2.7% | 45 | **1.5-10.3%** | **25-180** |
|  | CD8/CD3/CD62L-/CD45RA- | 5.8% | 96 | **1.1-9.2%** | **24-175** |
|  | CD8/CD3/CD62L-/CD45RA+ | 2.6% | 43 | **0.7-7.8%** | **11-172** |
|  |  |  |  |  |  |
| **B cell subsets** | CD20 | 14.6% | 242 | **3.0-19.0%** | **59-329** |
|  | CD19 | 14.6% | 242 | **3.3-19.3%** | **61-321** |
|  | CD20/CD27 | 3.2% | 53 | **0.8-3.6%** | **12-68** |
|  | CD20/CD38 | 12.9% | 214 | **1.2-17.6%** | **30-282** |
|  | CD20/CD10 | 3.7% | 62 | **0.6-6.0%** | **11-127** |
|  | CD21/CD10 | 3.1% | 51 | **0.2-4.4%** | **3-82** |
|  | CD20/IgM-/CD38++ | 0.0% | 0 | **0-0.1%** | **0-2** |
|  | CD20/IgM+/CD10+ | 2.2% | 37 | **0.5-5.3%** | **9-120** |
|  | CD20/CD38+/CD10+ | 2.3% | 38 | **0.5-5.9%** | **11-112** |
|  | CD20/CD27/IgM+ | 1.9% | 32 | **0.3-2.5%** | **6-50** |
|  | CD20/CD27/IgM- | 1.0% | 17 | **0.3-2.2%** | **6-43** |
|  | CD19/CD21 low/CD38 low | 9.0% | 295 | **0.1-1.8%** | **1-37** |
|  |  |  |  |  |  |
| **NK cells** | CD16+orCD56+/CD3- | 11.6% | 193 | **6.2-34.6%** | **126-729** |
|  | CD16+orCD56+/CD3+ | 7.4% | 123 | **2.2-12.4%** | **29-299** |

**Supplemental Table 2: Anti-cytokine autoantibody screening in our patient with POIKTMP.**

|  | **Patient** | **Mean +3SD of healthy controls** |
| --- | --- | --- |
| IFNγ | 267 | 904.2 |
| IFNα | 79 | 662.2 |
| IFNβ | 58 | 874.1 |
| IFNω | 202 | 1478.1 |
| GM-CSF | 88 | 364.73 |
| IL-1α | 278 | 1510.7 |
| IL-12p70 | 58 | 829.8 |
| IL-17A | 168 | 1053.5 |
| IL-17F | 171 | 727.3 |
| IL-22 | 139 | 1033.7 |

Shown are mean fluorescence intensity values. IFN, interferon; GM-CSF, granulocyte-macrophage colony stimulating factor; IL, interleukin. SD, standard deviation. Healthy controls n = 79.

**Supplemental Table 3: Top 20 identified Phip-Seq-based targets with the highest Z-scores relative to 126 healthy controls in our patient with POIKTMP.**

| **Gene** | **Name** | **Z-score** |
| --- | --- | --- |
| **CRTAC1†** | Cartilage Acidic Protein | 172.047795 |
| **HTRA1†** | HtrA Serine Peptidase 1 | 155.892447 |
| **ZNF7** | Zinc Finger Protein 7 | 110.806788 |
| **RHOV** | Ras-Homologue Family Member V | 110.751609 |
| **LINC02904** | Long Intergenic Non-Protein Coding RNA 2904 | 103.492419 |
| **CNTNAP2** | Contactin Associated Protein 2 | 99.705392 |
| **SLITRK6** | SLIT And NTRK Like Family Member 6 | 99.263212 |
| **PPIB** | Peptidylprolyl Isomerase B | 89.503796 |
| **SHANK2†** | SH3 And Multiple Ankyrin Repeat Domains 2 | 87.935881 |
| **TXNDC5** | Thioredoxin Domain Containing 5 | 76.035947 |
| **B3GAT1** | Beta-1,3-Glucuronyltransferase 1 | 76.013495 |
| **TMEM114** | Transmembrane Protein 114 | 67.423902 |
| **CNOT6** | CCR4-NOT Transcription Complex Subunit 6 | 67.240833 |
| **TSHZ3†** | Teashirt Zinc Finger Homeobox 3 | 66.066001 |
| **PHC3** | Polyhomeotic Homolog 3 | 65.524993 |
| **IVL†** | Involucrin | 60.989276 |
| **LOC645202** | Golgin A6 Family-Like | 59.185762 |
| **ZNF705A** | Zinc Finger Protein 705A | 55.674871 |
| **LOC105375297** | Uncharacterized LOC105375297 | 55.537955 |
| **TAGAP†** | T Cell Activation RhoGTPase Activating Protein | 55.10958 |

†Indicates that the target has tissue-level expression overlapping with our patient’s organ-specific disease manifestations.

**Supplemental Figure 1: Pedigree of our patient with POIKTMP.**


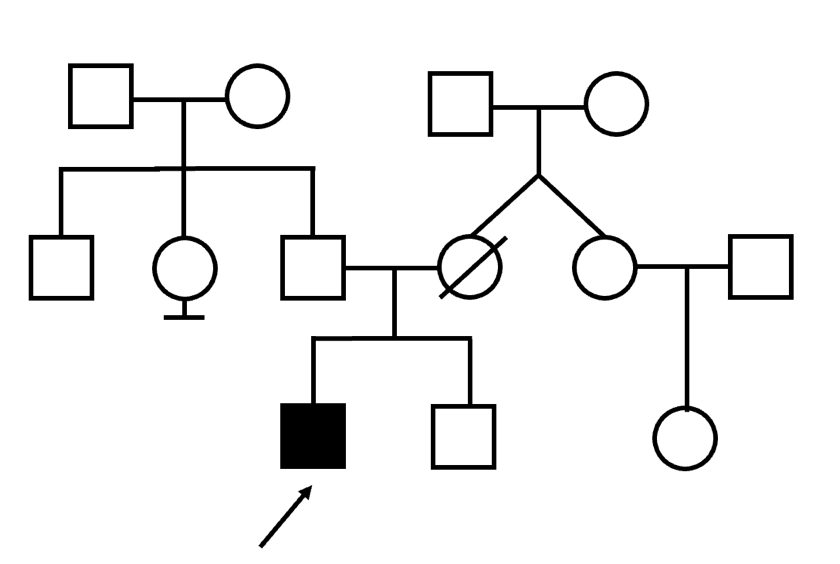


**Supplemental Figure 2: Axial views from non-contrast chest computed tomography.
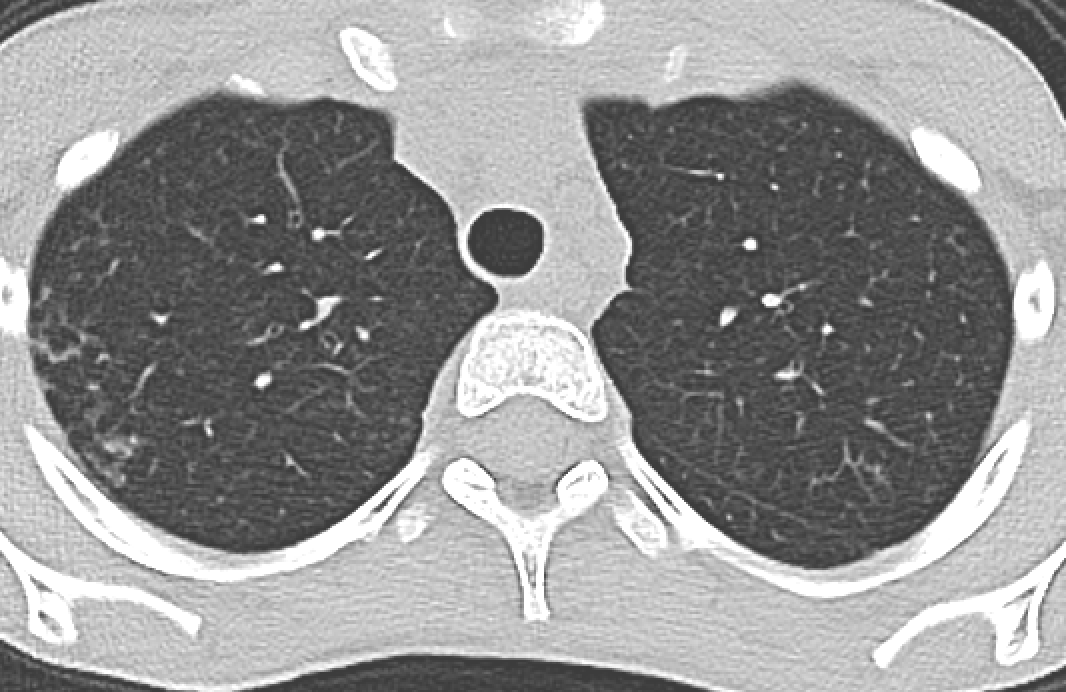
**
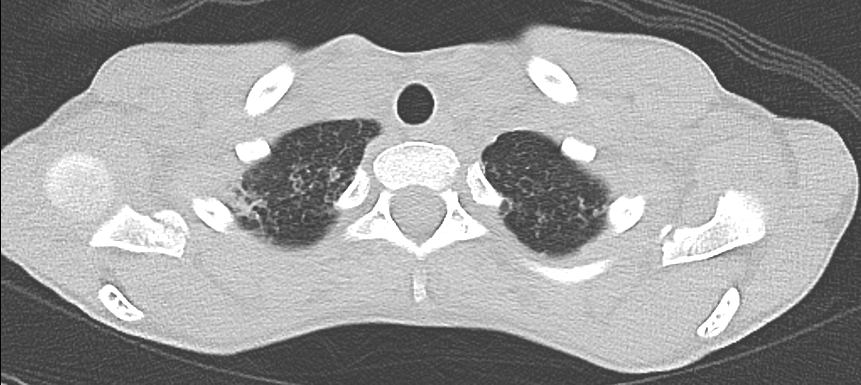
**
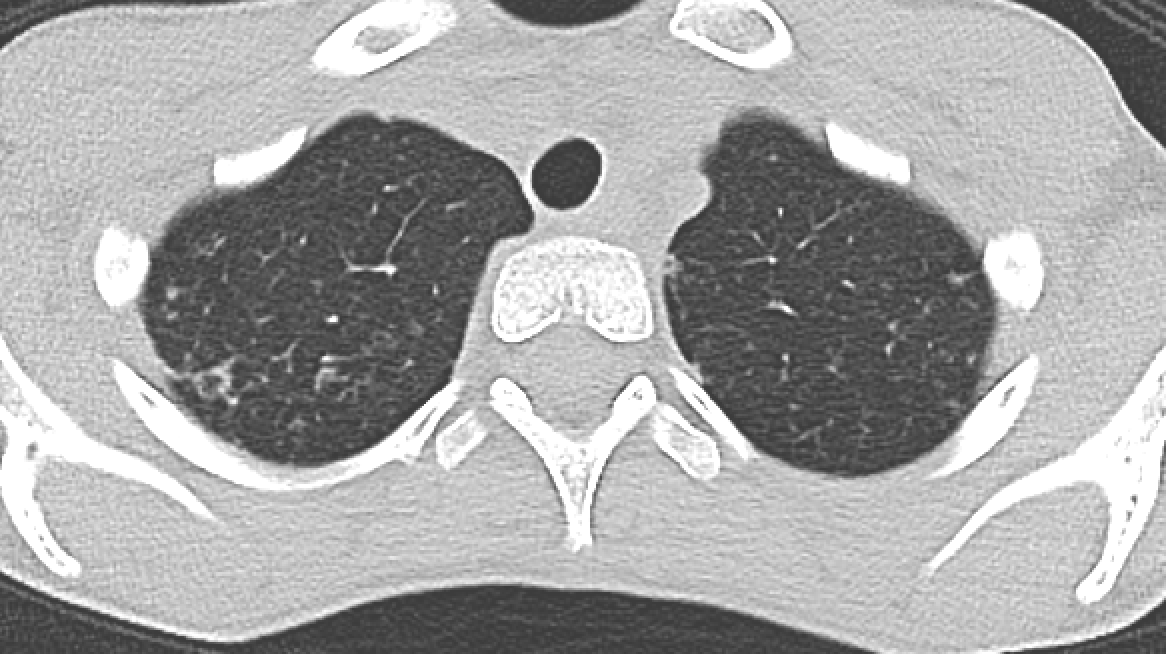
**
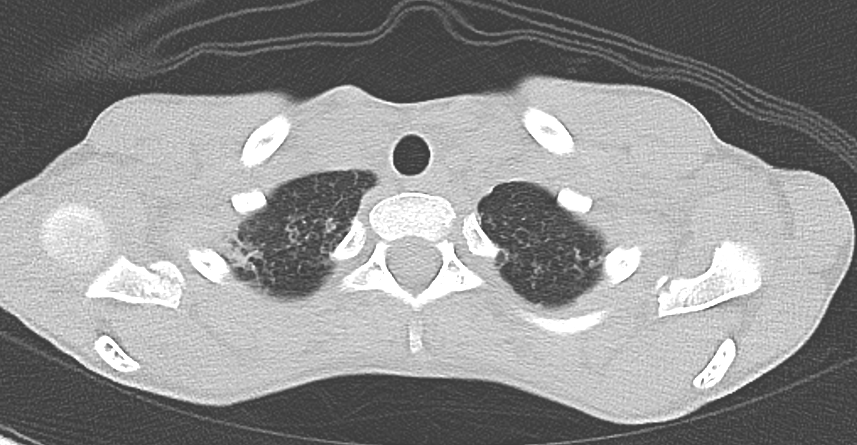


**Supplemental Figure 3**: **Immunophenotyping of T-lymphocytes in the peripheral blood.**


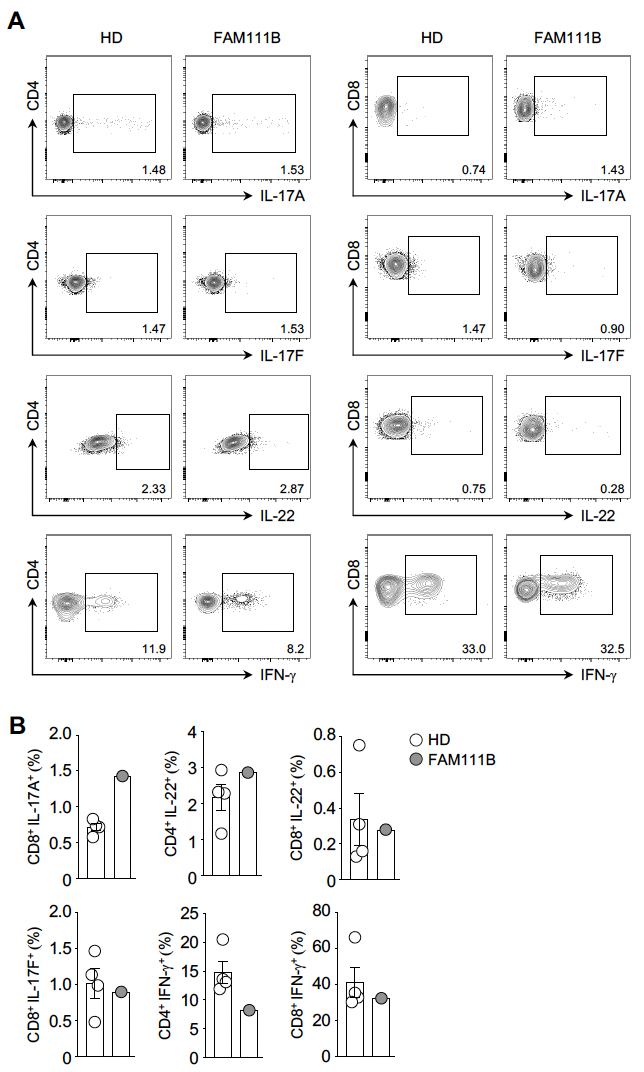


A) Representative contour plots of IL-17A, IL-17F, IL-22, and IFN-γ expression within CD4^+^ and CD8^+^ T cells in the peripheral blood from a healthy donor (left) and our patient (right). B) Frequencies of IL-17A^+^ and IL-17F^+^ within CD8^+^ T cells and IFN-γ^+^ and IL-22^+^ within both CD4^+^ and CD8^+^ T cells in the peripheral blood from our patient and four healthy donors.

**Supplemental Figure 1: Gating strategy for the flow cytometric immunophenotyping of peripheral blood T-lymphocytes.**
